# Visual Attention and Linguistic Preferences in Time-Telling: A Cross-Linguistic Study of German, Czech, and Russian Speakers

**DOI:** 10.1007/s10936-026-10234-z

**Published:** 2026-06-01

**Authors:** Elena Panfilova, Barbara Mertins

**Affiliations:** TU Dortmund, Dortmund, Germany

**Keywords:** Seeing for speaking, Language production, Time telling, Relative expression, Absolute expression

## Abstract

This study investigates how speakers’ initial fixations on analog clock regions (minute vs. hour hand) align with the production of relative and absolute time expressions in German, Czech, and Russian. Building on previous research on linguistic preferences in time‑telling, we tested whether fixation patterns primarily reflect syntactic word order, language‑specific expression preferences, or the interaction of both factors. Eye‑tracking data revealed broadly similar fixation patterns across languages for relative expressions: all groups tended to fixate first on the minute hand, consistent with syntactic structure and incremental planning models of language production by Levelt (Speaking: From Intention to Articulation. The MIT Press, Cambridge, 1989). In contrast, different patterns emerged for absolute expressions. Russian speakers showed a significantly stronger hour-first fixation pattern, consistent with the syntactic structure of absolute time expressions (“hour before minute”). German and Czech participants, however, distributed their initial fixations almost evenly across both areas of interest (minute‑hand and hour‑hand regions), suggesting that their strong habitual preference for relative expressions may attenuate syntactic ordering effects when producing less familiar absolute constructions. We interpret these findings as indicating that visual attention during speech production is shaped not only by syntactic structure (i.e., the structural order of time expressions such as hour-first vs. minute-first) but also by habitual language use. Our results highlight how cognitive processes such as visual attention interact dynamically with language use across cultural contexts.

## Introduction

How people integrate visual information with linguistic structures during speech production is a central question in psycholinguistics. Eye-tracking studies have consistently demonstrated that speakers’ gaze patterns closely follow the sequence of spoken elements, reflecting how visual cognition and linguistic planning interact during conceptualization (Gleitman et al., 2007; Griffin and Bock 2000; Griffin 2004a, b; Brown-Schmidt and Konopka 2008; Brown-Schmidt and Tanenhaus 2006). These findings have been interpreted as evidence of a causal relationship between language production and visual cognition, suggesting that the linguistic information required to produce an utterance can influence the programming of eye movements. This relationship has also been observed in experiments on time telling, where speakers must process clock hands visually before producing utterances describing the time (Bock et al., 2003; Hatzidaki et al., 2014; Kuchinsky et al., 2011). When processing visual information from the clock, speakers of various languages such as English or Russian must choose between two systems of time expression: an absolute system mentions the hour first followed by minutes (two-twenty) and a relative system mentions minutes first (twenty after two). These differences provide an ideal framework for investigating how linguistic structure shapes visual attention during conceptualization. Previous research on time-telling demonstrated that linguistic preferences for absolute or relative expressions vary across languages (Bock et al., 2003; Douglas et al., 2003; Hatzidaki et al., 2014; Kuchinsky et al., 2011; Panfilova & Mertins, 2026) and affect fixation patterns during time-telling tasks (Bock et al., 2003, 2004). These results support Slobin’s “thinking-for-speaking” theory (1996) and its extension, the “seeing-for-speaking” hypothesis, which posits that linguistic preferences and structures shape both attentional processes during communication tasks and memory encoding strategies (Bock et al., 2003; Kuchinsky et al., 2011; Mertins, 2018; Papafragou et al., 2008; v. Stutterheim et al., 2012; Schmiedtová, 2011).

While previous research has focused largely on instructed tasks involving Dutch, American English, or bilingual English-Greek speakers’ behavior during time telling (Bock et al., 2003; Hatzidaki et al., 2014), less is known about how cross-linguistic differences influence natural fixation patterns when no constraints are imposed on expression type. To test predictions derived from the “thinking-for-speaking” and “seeing-for-speaking” frameworks, our study examines whether speakers’ visual attention patterns during time-telling reflect the structural order of the expressions they produce, language-specific linguistic preferences (that is, habitual tendencies toward particular expression types), or the interaction between these two factors. Specifically, we asked the following research question: Do speakers’ eye-fixation patterns when reading time from analog clocks primarily reflect the structural order of the time expression they choose (e.g., hour-first for absolute vs. minute-first for relative expressions), broader linguistic preferences for particular temporal expression types (absolute vs. relative), or the interplay between these two influences? Building on prior research and relevant theoretical models, we hypothesize that speakers’ linguistic preferences for specific time expression types shape how visual information from clock hands is integrated during conceptualization. This integration process reflects the interplay between visual attention and language-specific planning mechanisms predicted by the “seeing-for-speaking” framework.

### Linguistic Preferences and Visual Attention During Time-Telling

Previous research shows that both linguistic preferences for relative versus absolute time expressions and structural differences between these expression types play a crucial role in guiding visual information processing during language production tasks such as time-telling (Bock et al., 2004). Eye-tracking studies have consistently shown that gaze patterns closely align with the linguistic elements being encoded into speech, reflecting an intricate relationship between perception, cognition, and language planning. Griffin and Bock (2000) investigated this relationship by using eye-tracking during spontaneous sentence production to examine how event comprehension relates to eye fixations on different elements of picture stimuli. The study was conducted with native American English speakers. It was found that speakers’ eye movements shifted systematically to each component of the depicted event, with each fixation preceding the corresponding part of the utterance by approximately 800 milliseconds. Importantly, the initial fixation typically aligned with the first word of the utterance, suggesting that gaze patterns are tightly coupled with linguistic planning processes. This finding highlights that sentence production influences visual focus beyond mere perceptual salience, emphasizing attentional processes are shaped by the goal of forming a coherent sentence.

In a study on message formulation during speech production, Brown-Schmidt and Konopka (2008) observed that speakers’ gaze patterns closely aligned with both syntactic structure and conceptual requirements during speech planning. In their visual‑world eye‑tracking experiment, English speakers fixated size‑contrast objects significantly earlier relative to noun‑phrase onset than Spanish speakers, approximately 480 ms before NP onset in English compared to about 100 ms in Spanish. This timing difference reflects cross‑linguistic variation in incremental planning: because English places adjectives before nouns (e.g., *the small butterfly*), speakers must plan adjective information earlier than Spanish speakers, whose post-nominal adjectives (e.g., *la mariposa pequeña*) allow later integration of size information. This tight temporal link between eye movements to relevant entities and subsequent linguistic expressions supports Levelt’s model of language production (1989), which proposes that conceptualization involves selecting task-relevant features incrementally based on lexical-syntactic requirements specific to each language.

Extending these insights from sentence processing to the domain of time-telling, Bock et al. (2003) examined whether similar relationships hold in time-telling tasks involving Dutch and U.S.-American English speakers. To investigate overall preferences for specific expression types in both language groups, they collected data from U.S.-American (*n* = 144) and Dutch (*n* = 144) students using paper-and-pencil questionnaires. Results showed that U.S.-American students used absolute expressions almost exclusively for both digital and analog time displays, whereas Dutch undergraduates preferred relative expressions across both stimulus types. In a subsequent eye-tracking experiment within the same study (Experiment 2), participants read times from either digital or analog clocks while their eye movements were tracked. They were explicitly instructed to produce either absolute expressions (ten twenty-five) or relative expressions (twenty-five past ten), with half of the speakers in each language group encouraged to use one type and the other half the opposite type. Key findings included that eye movements mirrored the linguistic structure of time expressions: participants producing absolute expressions fixated first on the hour hand before shifting to minutes, matching the order of verbal expression, whereas producing relative time expressions this pattern was reversed by fixating minute information first. Interestingly, two U.S.-American English speakers deviated from the typical fixation pattern by consistently fixating on the hour hand first even when producing relative expressions. Although this observation involved only two individuals (one in Experiment 2 and another in Experiment 3), Bock et al. speculated that such deviations might reflect subtle influences of linguistic preference on visual information uptake. This interpretation suggests that even within highly controlled tasks, individual linguistic habits may modulate how speakers coordinate visual attention and speech planning (Bock et al., 2003, p. 682). In addition to the fixation patterns, Bock et al. also reported differences between analog and digital displays: responses to digital clocks were generally faster than those to analog ones, with a larger advantage for absolute than for relative expressions. For analog clocks, relative expressions were produced faster than absolute ones, a pattern observed among Dutch but not American English speakers. Although the sample size in Bock et al.‘s (2003) study was relatively small (*n* = 8 for each language group), their findings provide valuable initial insights into the interplay between linguistic preferences and visual processing during time-telling tasks. The results not only suggest that linguistic structures guide visual focus during time-telling but also point to potential influences of language-specific usage patterns: for instance, as discussed above, which may reflect the broader preference for absolute expressions among English speakers. Bock et al. (2004) explored whether task-induced priming had influenced earlier findings. American English speakers were asked to read times from analog clocks without specific instructions. The most responses reflected a strong preference for absolute expressions, a characteristic already observed among American English speakers (~ 89% preference for analog displays; Bock et al., 2003). Crucially, regardless of expression type produced (i.e. absolute or relative), participants tended to fixate first on the hour region – further supporting claims that linguistic preferences shape initial uptake of visual information more strongly than syntactic constraints alone.

Expanding this research across languages with different syntactic structures, Hatzidaki et al. (2014) studied Greek-English bilinguals (*n* = 40) alongside English monolingual participants (*n* = 20). In their study, participants produced only relative time expressions while viewing digital or analog clocks. Importantly, the structure of relative expressions differs between the two languages: in English, minute information precedes hour information (e.g., “ten past nine”), whereas in Greek it follows the hour (e.g., Είναι εννιά και δέκα, “It’s nine and ten”). The analysis of the first fixation generally revealed that speakers’ eye movements reflected the structure of the time expression they are producing. However, this effect was significant mainly with digital clocks. In contrast, when viewing analog clocks, a pattern emerged where all groups initially focused on the hour region regardless of language spoken or expression type produced. A finding interpreted by the authors as a universal visual tendency driven by the greater perceptual complexity of analog displays. Yet for Greek bilinguals, this fixation pattern could also stem from linguistic influences rather than purely perceptual ones, because Greek relative time expressions align in word order with absolute expressions by presenting hour information before minutes (*Είναι εννιά δέκα*, ‘It’s nine ten’). This alignment may naturally prompt Greek speakers to focus on the hour region first. For British English monolinguals in this study, interpretation remains limited due to missing data on their time-telling preferences, leaving open whether gaze patterns were guided primarily by syntax or broader linguistic preferences.

Taken together, syntactic structure determines fixation order during speech planning, but long‑term language habits can modulate or even override these structural effects. This interplay between syntax and preference provides the foundation for our cross‑linguistic comparison of German, Czech, and Russian speakers. Building on these insights from previous cross‑linguistic research, the present study focuses specifically on German, Czech, and Russian, three typologically distinct yet culturally connected languages, to examine how linguistic structure and habitual expression preferences jointly shape visual attention during time telling.

### Cross-Linguistic Dimensions: German, Czech, and Russian

The selection of these languages allows for meaningful comparisons across linguistic dimensions. Czech and Russian, both part of the Slavic language family, share numerous lexical, grammatical, and structural similarities despite belonging to different subgroups (East Slavic for Russian and West Slavic for Czech). At the same time, Czech and German are part of the Central European *Sprachbund* (Nekula, 2003), a linguistic area shaped by geographical proximity and historical interactions, which has led to notable structural and lexical overlaps between these typologically distinct languages. Research suggests that German influence extends beyond vocabulary into Czech grammar (Havránek & Fischer, 1965; Berger, 2009) and even conceptual preferences (Mertins, 2018).

To illustrate how these linguistic dimensions manifest in time-telling structures across the three languages, Table 1 presents examples of absolute and relative expressions with literal English translations.

**Table 1:** Relative and absolute time expressions in German, Czech and Russian with literal English glosses

Time	Expression type	German + English glosses	Czech + English glosses	Russian + English glosses
2:35	Relative	*fünf nach halb drei* (‘five past half three’)	*půl třetí a pět minut* (‘half three and five minutes’) *za deset minut tři čtvrtě na tři* (‘in ten minutes three quarters to three’)	*тридцать пять минут третьего* (‘thirty-five minutes of the third [hour]’) *без двадцати пяти три* (‘without twenty-five [minutes] three’)
2:40	Relative	*zwanzig vor drei* (‘twenty to three’)	*půl třetí a deset minut* (‘half three and ten minutes’) *za pět minut tři čtvrtě na tři* (‘in five minutes three quarters to three’)	*без двадцати три* (‘without twenty [minutes] three’)
2:35	Absolute	*zwei Uhr fünfunddreißig* (‘two hour thirty-five’)	*dvě třicet pět* (‘two thirty-five’)	*два тридцать пять* (‘two thirty-five’)
2:40	Absolute	*zwei Uhr vierzig* (‘two hour forty’)	*dvě čtyřicet* (‘two forty’)	*два сорок* (‘two forty’)

Previous cross-linguistic research on time-telling preferences among native speakers of German, Czech, and Russian provides important baseline information on habitual linguistic tendencies relevant to the current eye‑tracking study (Panfilova & Mertins, 2026). In that study, native speakers of all three languages were tested using paper-and-pencil questionnaires with analog clock displays (*N* = 396; *n* = 132 per language). Participants wrote out the depicted times in words without being instructed on expression type. This design allowed for a reliable estimation of linguistic preferences based on a large sample size and balanced stimulus set covering eleven five-minute intervals per hour. The results showed strong preference for relative expressions among German (92%) and Czech (86%) speakers whereas Russian speakers demonstrated a more balanced distribution between relative and absolute forms (64% vs. 36%). Given these differences in linguistically shaped preferences for relative expressions for German and Czech speakers versus more balanced for native Russian speakers, we expect variations in fixation patterns that are influenced not primarily by linguistic structure but by the preferred type of time expression. 

## The Present Study

### Research Question and Hypotheses

This experiment investigated whether linguistic preferences in time-telling influence how native German, Czech, and Russian speakers process visual information from analog clocks. Specifically, we aimed to explore how language-specific syntactic structures and cultural preferences for certain types of time expressions shape eye fixation patterns during speech production.

We formulated our following research question: Do speakers’ initial fixations during time-telling reflect (a) the syntactic word order of their chosen time expression, (b) language-specific preferences for particular expression types, or (c) an interaction between these two factors?

If only linguistic preferences play a role, fixation patterns should correspond to the dominant expression type used in each language – minute-first fixations for languages that favor relative expressions (German and Czech). In contrast, if only structural order determines gaze behavior, speakers should consistently fixate first on the element that appears first in the syntactic sequence of their produced utterance – hour-hand fixations for absolute expressions and minute-hand fixations for relative ones. However, if both linguistic preferences and structural order jointly influence visual attention, speakers’ fixation patterns are expected to reflect an interaction between these factors rather than a single dominant effect. Accordingly, we formulated the following hypotheses to capture this predicted interaction:

1. Given strong preferences for relative expressions in German (92%) and Czech (86%), speakers of these languages will exhibit initial minute-hand fixations while producing relative expressions because structural order coincides with the preferred expression type.
2. For German and Czech speakers producing absolute expressions, we anticipate a more complex pattern. While their strong preference for relative expressions might predispose them toward initial fixations on the minute hand, syntactic structure (which requires hour-first ordering in absolute expressions) may counterbalance this tendency.

3. Because Russian speakers show a more balanced distribution between relative and absolute expressions (64% relative), we expect their fixation patterns to align more closely with syntactic word order: hour-first fixations for absolute expressions and minute-first fixations for relative expressions.

### Method

#### Participants

In the study, native speakers of German, Czech, and Russian were recruited (*N* = 117 in total). The recruitment and data collection period for this study began on 9 May 2023 and concluded on 20 April 2024. All participants were informed about the study’s aims, procedures, and their rights. Written informed consent was obtained from every participant before their involvement in the study. Additionally, we ensured that participants’ confidentiality and anonymity were maintained following data protection regulations. The students from the German and Czech groups took part in the study to earn credit for their classes. The Russian-speaking participants received a compensation of 10 euros for their participation in the study.

Depending on the native language, we formed three groups of participants:

1. German native speakers consisted of students at TU Dortmund in Germany (*n* = 40; f = 33), with ages ranging from 18 to 46 years (mean = 23; SD = 4,6).
2. Czech native speakers consisted of students at Charles University in the Czech Republic (*n* = 40; f = 32), aged between 19 and 47 years (mean = 22,7; SD = 5,3).
3. Russian-speaking participants consisted of Ukrainian and Russian refugees (*n* = 37; f = 29), aged between 18 and 68 years (mean = 43,8; SD = 14,3). All of them had fled to Germany following the onset of the Russian invasion of Ukraine. They were recruited in German A1 courses and indicated Russian as their mother tongue, which they spoke at home. Because this group was more demographically diverse and not limited to university students, we collected additional information on educational background: among the 37 participants, eleven did not hold a university degree; four of these were undergraduate students and enrolled in distance programs at Ukrainian universities at the time of testing, while the remaining seven had completed vocational training but were not pursuing higher education. This heterogeneity reflects the exceptional circumstances under which this group was recruited.

All participants reported normal or corrected-to-normal vision. We excluded two German participants because both of them indicated to be bilingual (Russian-German and Italian-German) and one Czech participant with a low tracking ratio. For the tracking ratio we chose the level of 75% as a benchmark for inclusion before the data analysis started (see, e.g., Riege et al., 2021 for comparison).

### Eye-Tracking Experiment

#### Materials

**Fig. 1:**
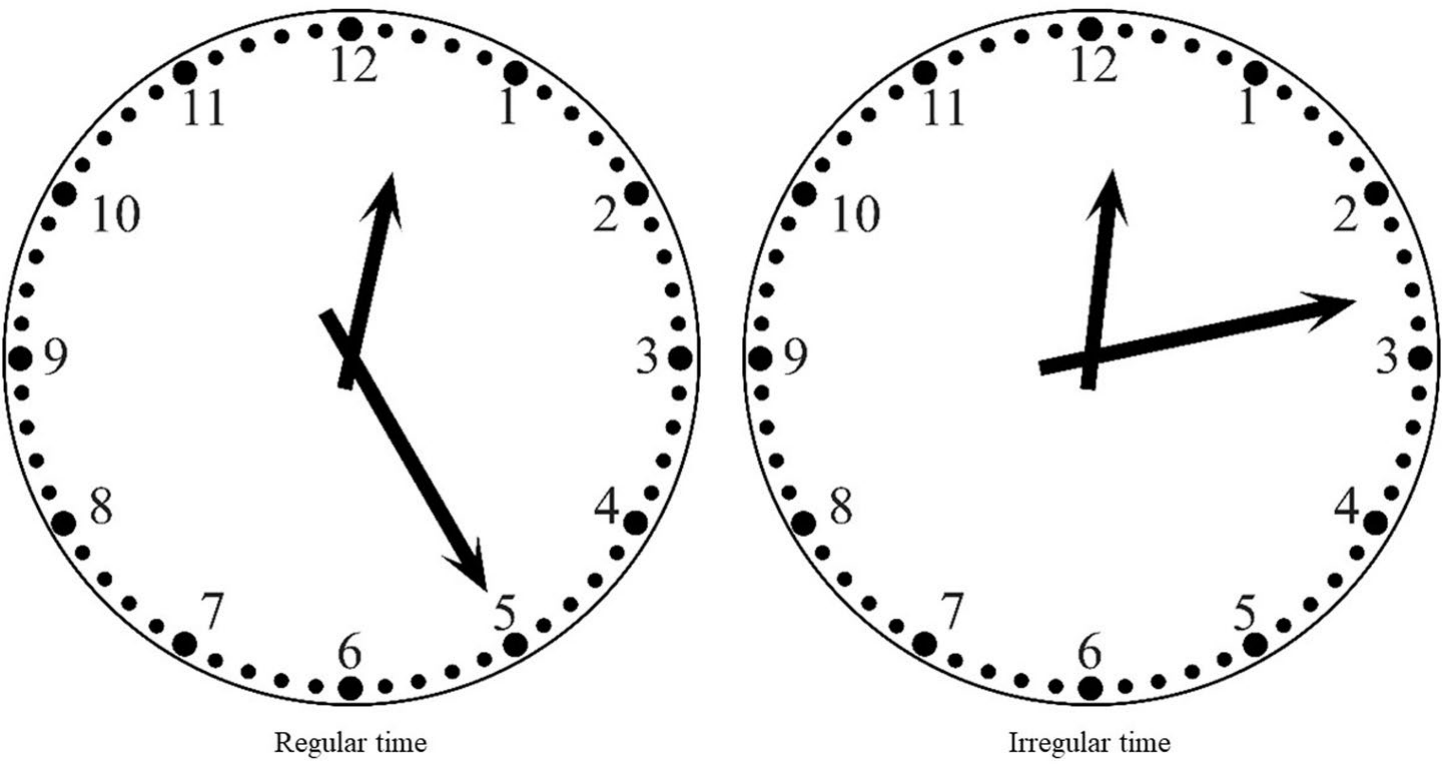
Examples of regular (left) and irregular clock faces (right) used in the study

The materials consisted of 192 different analog-format times. Of these, 144 images represented five-minute intervals within a 12-hour period from 12:00 to 11:55 (12 types with 12 observations each). These intervals are referred to as regular times. Additionally, 48 clock images depicted minute intervals (four per hour), referred to as irregular times. These irregular stimuli were chosen based on two criteria: (a) the hour and minute hands formed an angle of approximately 30°, ensuring that the areas of interest did not overlap; and (b) minute positions did not repeat across hours to maximize visual variety. All clocks were numbered, tick-marked, and featured parametric hour hands indicating smooth movement between hours as minutes progressed. Examples of both stimulus types (regular and irregular) are depicted in Fig. 1.

**Fig. 2:**
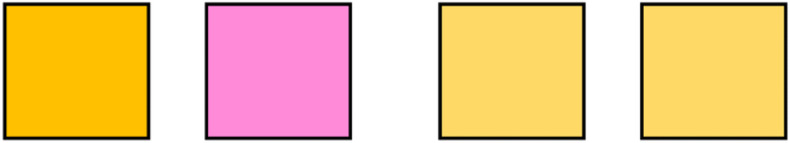
Examples of fillers with different colors (left) and identical colors (right) used in the study

In addition to the main stimuli, we incorporated 100 filler items into the experiment. These fillers consisted of images featuring two colored squares, with half displaying squares of different colors and the other half showing squares of the same color, and were included to provide brief variation and reduce fatigue during the experiment. The examples of fillers are shown in Fig. 2.

#### Equipment and Procedure

Participants were tested individually in a laboratory setting, conducted within a dimly lit, soundproof room. Testing occurred in front of a computer monitor (Dell, 40” with a 16:10 aspect ratio). The distance between the participant and the screen was approximately 67 cm. Eye movements were tracked using a remote SMI RED eye-tracker, operating at a sample rate of 60 Hz. Participants’ utterances were captured using the build-in stereo microphone of a Logitech HD Pro Webcam. For stimulus presentation and data acquisition, the SMI Experiment Center was utilized, which recorded eye movement data time-locked.

The stimulus size in pixels was 4627 × 4626 for clocks and 960 × 720 for fillers. The stimuli and the fillers were displayed “fit to screen,” resulting in identical on-screen size presentation across conditions.

A calibration procedure followed by validation was conducted for each participant before the experiment. Calibration was accepted if the average error was < 0.30° of visual angle and the maximum error was < 0.50°. After the calibration, written instructions appeared on the screen. One task involved identifying whether two squares on the screen shared the same color or differed in color, while the other task required naming the depicted time from the analog clock. Participants were not explicitly instructed on which type of time expression (relative or absolute) to use during the experiment. Consequently, participants were free to use any time expression they preferred.

**Fig. 3:**
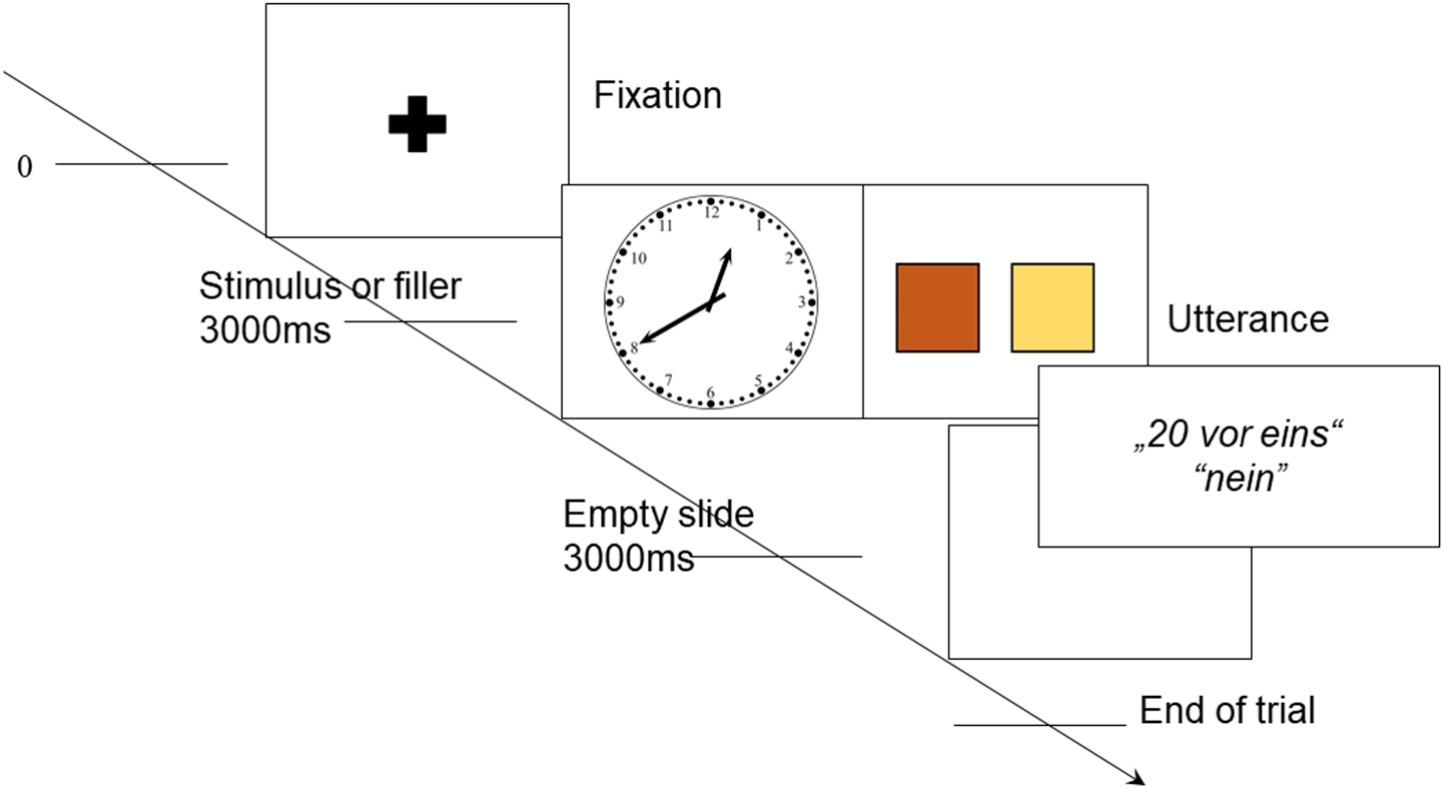
Sequence and timing of trial events (German example)

At the onset of each trial, a fixation cross was presented in the center of the display. The following stimulus was activated by 1000 msec of uninterrupted fixation on the cross. Each stimulus was presented for 3000 msec followed by a blank screen displayed for an additional 3000 ms, leaving speakers enough time to plan, start, and finish their utterance. Participants were instructed to name the time as soon as they recognized it, without waiting for the blank screen. All stimuli were presented in randomized order. The main experimental session lasted about 30–35 min. Figure 3 illustrates the sequence of trial events.

To optimize the efficiency and effectiveness of the main experiment, each participant first took part in a short test run before the main session. This testing session consisted of eight clock pictures (full hour, quarter, half, three-quarters times, :10 and :50-minute times, and two irregular times, such as 7:13; 10:43) and two fillers (one same-colored and the other differently-colored squares). The test run followed the same procedure as the main experiment, but the stimuli used in the test run were later excluded from the analysis. The experiment was conducted in the native language of the participants: German, Russian, or Czech.

#### Eye-Gaze Analysis

The areas of interest (AoIs) were predetermined before the recordings. For each clock picture, we designated two AoIs, each spanning a 30-degree angle around the hour and minute hands, respectively. During each trial, the region relevant to the hour hand was determined by its position, and similarly for the minute hand. The AoIs were not marked for the participants during the stimulus presentation. The initial fixation, located at the screen’s center due to the preceding fixation cross, was omitted from the analysis, along with fixations outside the time-relevant AoIs (which were categorized as “other”). 22 clock times with completely or partially overlapping hour and minute AoIs (e.g., 0:05, 1:05, 1:10, 2:10, 2:15, etc.) were omitted from the gaze analysis but retained for the speech analysis.

#### Speech Analysis

The expressions were transcribed and coded during the experimental session. Utterances were classified as absolute when the hour information was followed by the minute information, and as relative when the words ‘quarter’, ‘half’, or the minute number preceded the hour number. All whole‑hour trials were excluded from the analysis because they cannot be classified as relative or absolute expressions. All erroneous responses were excluded from the analysis. However, non‑standard responses that were correct in principle but deviated from conventional time‑telling formulations (e.g., *Vierzig nach drei*, ‘forty past three’) were coded according to criteria provided above and treated as correct answers. These criteria were consistently applied across all three languages.

#### Validation

To evaluate the reliability of the coding process, three additional coders with native proficiency in the analyzed languages independently coded 10% of the data, comprising a total of 400 utterances per language. The coding followed the predefined rules (see Speech analysis). Subsequently, intercoder reliability was assessed using Cohen’s kappa index, calculated by dividing the number of actual matches by the total number of utterances. The resulting Cohen’s kappa value between the coders was 0.99 for German and Czech and 0.98 for Russian, indicating an “almost perfect” level of agreement (Landis & Koch, 1977; McHugh, 2012).

### Supplementary Survey: Participants’ Background and Clock-Usage

To complement the eye‑tracking data, participants completed an online survey designed to collect demographic information, linguistic background details, and data on clock‑usage habits. The survey was implemented via LimeSurvey (LimeSurvey GmbH, n.d.) and administered partly before and partly after the experimental session.

The first section gathered general demographic information including age, handedness, gender, vision quality, education level, and color blindness. To control for potential effects of mathematical ability on time‑reading skills (Andersson, 2008; Burny et al., 2011), participants also indicated whether they experienced difficulties with mathematics or dyscalculia.

Questions on linguistic background ensured comparability across groups by including only monolingual speakers who had been exposed to one language before school age. This definition was applied consistently across all groups. Although most Russian-speaking participants were technically Russian-Ukrainian bilinguals, all reported acquiring Ukrainian only upon entering school (around age of seven) rather than in early childhood. Given that Russian and Ukrainian share identical structural means for expressing time relations (absolute vs. relative expressions), systematic cross-linguistic influence is unlikely to have substantially affected performance.

Given the increasing prevalence of digital devices and declining familiarity with analog clocks among younger adults (McDaniel et al., 2024), we also collected brief questionnaire data after the eye‑tracking session to control for potential effects of analog exposure across groups. Participants completed a short-structured survey about their everyday clock usage. The questionnaire was originally designed to explore potential differences in exposure to analog clocks across language groups. However, the results revealed very similar patterns among all participants. These findings are briefly discussed in the Discussion section as supporting evidence that group differences in fixation behavior are unlikely to be driven by variations in everyday experience with analog clocks.

## Results

The subsequent section presents the findings in relation to the research question and hypotheses formulated in the Section “Research question and hypotheses”. The statistical analysis was performed using R Statistical Software 4.5.1. To address our research question, we began with a descriptive analysis of fixation patterns across German, Czech, and Russian speakers.

The descriptive data revealed broadly similar fixation patterns across languages, with minor variations in relative frequencies. German and Czech speakers showed a slight preference for the minute-hand region over the the hour-hand region in their initial fixations (53% in Czech and 60% in German), whereas Russian participants exhibited a comparable distribution with a marginal tendency toward hour-first initial fixations (53%). These small proportional differences do not suggest distinct cross‑linguistic strategies but rather indicate overall similarity in initial visual attention during time‑telling tasks.

Analysis of linguistic data revealed additional cross-linguistic variations. German speakers confirmed expectations by predominantly producing relative expressions (84%), consistent with prior studies. Russian speakers displayed a more balanced distribution, producing 53% relative expressions and 47% absolute expressions. Czech speakers, however, deviated from previous findings (Panfilova & Mertins, 2026). Unlike German but similar to native Russian speakers, they produced nearly equal proportions of relative (48%) and absolute (52%) expressions.

**Fig. 4:**
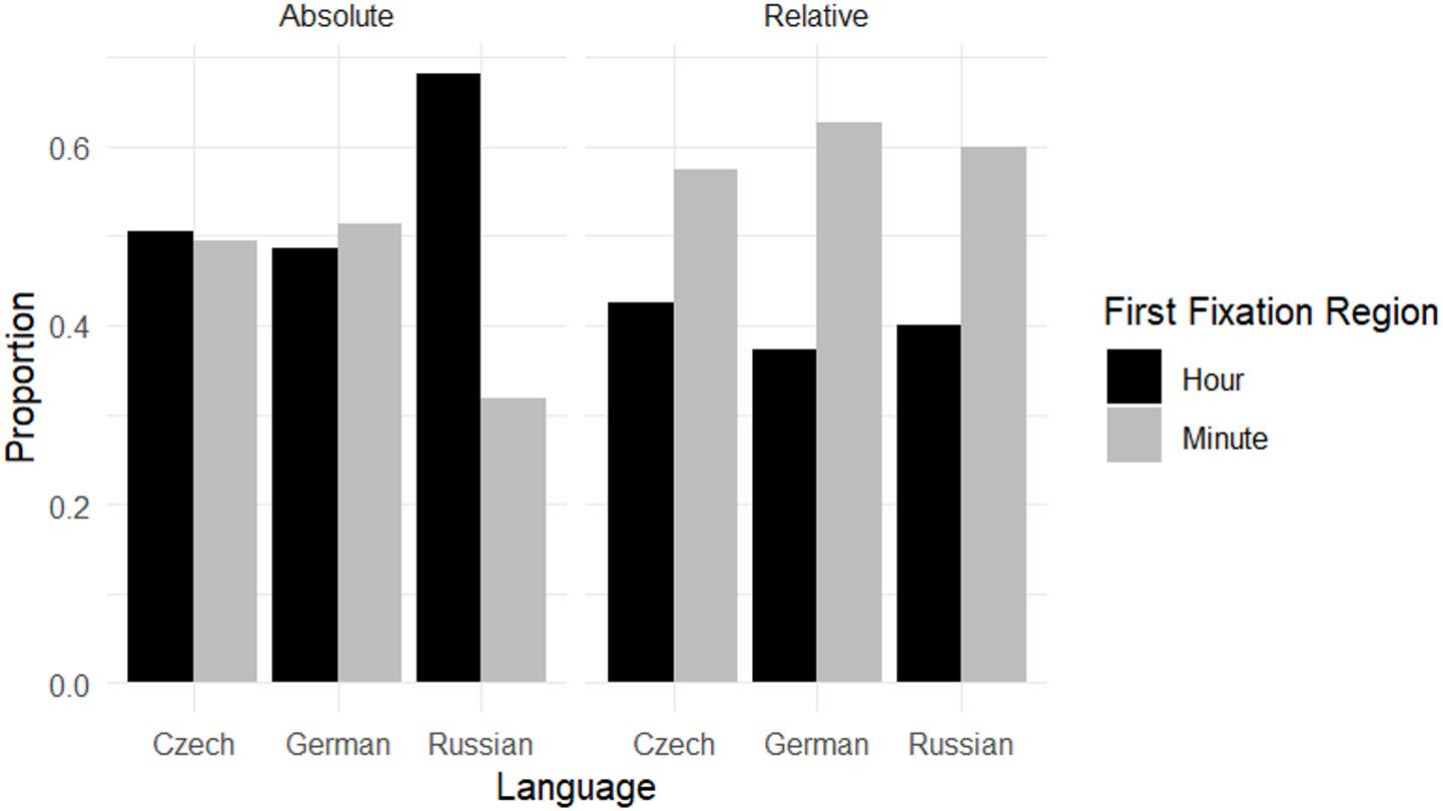
Proportion of fixations on the hour and minute region based on the type of time expression

In the next step, we analyzed how initial fixation regions corresponded to the subsequent type of time expression produced. The results are shown in Fig. 4.

When producing relative time expressions, participants across all three languages tended to fixate first on the minute-hand region (Czech ≈ 57%, German ≈ 63%, Russian = 60%), consistent with the syntactic structure of relative expressions. For absolute expressions, fixation patterns were more evenly distributed among Czech and German speakers, who showed similar proportions for hour‑first and minute‑first fixations (≈ 50% each). In contrast, Russian participants displayed a higher proportion of hour‑first fixations (≈ 67%).

These findings highlight how linguistic preferences interact differently with gaze behaviors depending on both language group and type-specific constraints within spoken utterances.

To assess how time-telling preferences influenced initial fixations on AoIs their relation to expression type and language, we conducted regression analyses using a Generalized Linear Mixed Model (GLMM) with maximum likelihood parameter estimation (Laplace Approximation). A binomial response variable with a logit link function was modeled, using German as a reference level across the dataset. Statistical analyses were performed in R using the ‘**glmer()**’ function of the ‘**lme4**’ package (Bates et al., 2015).

Descriptive analysis revealed cross-linguistic differences in first fixation regions and expression types (see Fig. 4). To formally test our a priori hypotheses, we fitted a GLMM with first fixation in AoI as the dependent variable. Fixed effects included language (German, Czech, Russian), expression type (relative vs. absolute), stimulus type (regular vs. irregular), and age, as well as the interaction between language and expression type. Language was coded using treatment contrasts with German as the baseline level. German was chosen because it represents a typologically distinct non‑Slavic language within our dataset, providing a meaningful point of comparison for Czech and Russian speakers. Expression type was dummy‑coded with absolute expressions as the reference level; coefficients for relative expressions therefore represent differences compared to this baseline condition. Random intercepts were specified for stimuli, and a random slope for expression type was included at the participant level.

A Likelihood Ratio Test (LRT) comparing models with and without interaction revealed a significant improvement in fit when including interaction effect (χ^2^ = 92.329, *p* < .001), confirming that fixation patterns differed across languages depending on expression type.

Given the wide age range of participants, particularly within the Russian group, and prior evidence that time-telling preferences may vary across generations (McDaniel et al., 2024), we additionally tested an exploratory Age * Language interaction to examine possible differential age effects across language groups. The extended model did not significantly improve fit compared to the original model without the interaction (χ²(2) = 5.16, *p* = .076), and neither interaction coefficient reached significance (*p* = .21 for Russian; *p* = .54 for Czech), indicating that age did not differentially modulate fixation patterns across groups. Age was retained as a fixed effect in the final model, whereas the non‑significant Age * Language interaction was excluded.

The equation for GLMM was as follows: First fixation in AoI ~ age + stimulus type + language * expression type + (1 + expression type | participant) + (1 | stimulus). The final model predicting first fixation location is summarized in Table 2.

**Table 2:** Output of Model applied to the whole data set

Predictors	Dependent variable			
	First fixation in the AoI (minute)			
	**Estimate**	Standard error	z-value	*p*-value
*Fixed effects*				
(Intercept)	0.262	0.163	1.605	0.109
Expression type (relative)^1^	0.299	0.154	1.945	0.052
Age^2^	-0.065	0.074	-0.886	0.376
Russian^3^	-0.894	0.217	-4.114	< 0.001***
Czech^3^	-0.2	0.182	-1.102	0.27
Stimulus type	-0.133	0.124	-1.077	0.282
Russian*Expression type	0.783	0.217	3.605	< 0.001***
Czech* Expression type	-0.066	0.203	-0.323	0.746
	Variance		*SD*	Corr.
*Random effect*				
Stimulus (Intercept)	0.499		0.699	
Participant (Intercept)	0.33		0.575	
Expression type | participant	0.35		0.593	− 0.69

^1^Expression type was dummy‑coded with absolute expressions as the reference level

^2^Age was z-transformed (mean-centered and scaled by the standard deviation)

^3^Coefficients for “Language (Russian)” and “Language (Czech)” represent contrasts against German baseline; interaction terms indicate differences across expression types

*p < .05

**p < .01

***p < .001

Age did not significantly predict fixation behavior (*b* = − 0.065, *p* = .376), nor did stimulus type (*b* = − 0.133, *p* = .282). The main effect of expression type was not statistically significant (*b* = 0.299, *p* = .052), although relative expressions descriptively elicited more minute‑first initial fixations than absolute expressions.

Significant cross‑linguistic differences emerged. Overall, native Russian speakers were less likely than German speakers to fixate first on the minute hand (*b* = -0.894, *p* < .001). A significant interaction between language and expression type further indicated that this pattern differed across languages: Russian-speaking participants showed a stronger shift toward minute-first fixations when producing relative expressions compared with German speakers (*b* = 0.783, *p* < .001). In contrast, the interaction between language and expression type did not differ significantly between Czech and German speakers , indicating no reliable difference in how expression type influenced fixation patterns in these two groups.

Based on the significant interaction observed, we computed estimated marginal means (EMMs) from the fitted model to facilitate pairwise comparisons among language groups. EMMs provide model‑based predictions adjusted for other factors in the analysis, allowing for direct comparison of group differences on the logit scale (Lenth, 2024). We used EMMs to obtain additional contrasts not directly available from the model output (e.g., Czech vs. Russian) and applied Tukey adjustments to control for multiple testing.

EMMs indicated that for relative expressions German speakers showed the highest logit values (EMM = 0.495), followed by Russian-speaking (EMM = 0.383) and Czech participants (EMM = 0.229). For absolute expressions, native Russian speakers exhibited substantially lower logit values (EMM = -0.699) compared with both German (EMM = 0.195) and Czech participants (EMM = -0.005).

Pairwise contrasts confirmed significant differences between native Russian and German speakers (*b* = -0.894, *p* < .001) as well as between native Russian and Czech speakers (*b* = 0.694, *p* < .01). Within the Russian-speaking group, a significant difference was observed between relative and absolute time expressions (*b* = -1.083, *p* < .001), indicating a stronger shift toward minute‑first fixations during relative expression production, whereas absolute expressions were associated with hour-first fixations. No other pairwise comparisons reached significance. Further details of the estimated marginal means and relevant pairwise contrasts are presented in Table 3.

**Table 3:** Summary of the estimated marginal means and relevant pairwise contrasts for each language and expression type

Language	Expression type	emmean (logit)	Standard error	95% CI [LCL, UCL]^1^
German	Absolute	0.195	0.159	[-0.117, 0.507]
Russian		-0.699	0.158	[-1.010, -0.389]
Czech		-0.005	0.133	[-0.265, 0.255]
German	Relative	0.495	0.108	[0.282, 0.707]
Russian		0.383	0.151	[0.087, 0.679]
Czech		0.229	0.122	[-0.010, 0.467]
Selected pairwise contrasts				
Contrasts		Estimate (logit)		*p*-value
Russian vs. German (Absolute)		-0.894		< 0.001***
Czech vs. Russian (Absolute)		0.694		< 0.01**
Russian: Relative vs. Absolute		-1.083		< 0.001***

^1^CI = 95% confidence interval; LCL = lower confidence limit; UCL = upper confidence limit. Values are reported on the logit scale

*p < .05

**p < .01

***p < .001

## Discussion

This study investigated how initial fixations on areas of interest (the minute-hand and hour-hand regions) align with the production of relative and absolute time expressions across three languages: German, Czech, and Russian.

Our research question examined whether speakers’ initial fixations during time-telling reflect either (a) the syntactic word order of their chosen time expression, (b) language-specific preferences for particular expression types, or (c) an interaction between these two factors. Based on the previous finding on linguistic preferences in time-telling (Panfilova & Mertins, 2026) our hypotheses predicted minute-first fixations for German and Czech when producing relative expressions, as structural order coincides with the preferred expression type. For absolute expressions, we anticipated a more balanced pattern for these groups due to the interplay between habitual linguistic preference and syntactic structure. In contrast, for Russian speakers, we predicted hour‑first fixations in absolute expressions, reflecting alignment with syntactic word order. The results largely confirmed these expectations. Pairwise comparisons based on estimated marginal means (EMMs) indicated broadly similar fixation patterns across languages for relative expressions: all groups showed a tendency toward minute‑first fixations, and no significant differences were found between language groups (all ps > 0.05). This pattern is consistent with previous findings that gaze behavior reflects the syntactic structure of expression (Bock et al., 2003; Griffin & Bock, 2000; Brown-Schmidt & Tanenhaus, 2006; Brown-Schmidt & Konopka, 2008) and supports the language production model of Levelt (1989), which suggests that conceptual units are selected sequentially based on lexical-syntactic demands. We interpret these findings in light of Slobin’s “thinking-for-speaking” framework (1996). This interpretation also resonates with Schmiedtová’s (2011) extension of this idea, the “seeing-for-speaking” hypothesis, suggesting that linguistic preferences may shape how speakers allocate visual attention when conceptualizing messages. Furthermore, our results are consistent with Brown-Schmidt & Konopka’s research on incremental planning at lexically sized units, implying that even subtle syntactic differences might be reflected in how pre-linguistic concepts are linked to visual attention during language production tasks.

For absolute expressions, fixation behavior varied across languages: Russian speakers showed significantly stronger hour‑first fixation patterns than both German and Czech participants (*p* < .001 and *p* < .01), consistent with the syntactic structure of absolute time expressions (“hour before minute”). In contrast, German and Czech speakers exhibited no significant preference for either the hour or the minute AoI in their first fixation (ps > 0.05), suggesting that their strong habitual preference for relative forms may influence attentional strategies when producing less familiar absolute expressions. One plausible explanation lies in the overall linguistic preferences. Previous studies indicate that Germans and Czechs overwhelmingly favor relative expressions (92% in German and 86% in Czech; Panfilova & Mertins, 2026), which may influence their attentional strategies when producing less familiar absolute expressions. In contrast, Russian speakers exhibit a more balanced use of relative and absolute expressions, likely fostering stronger alignment between gaze behavior and syntactic structure during speech production. This finding aligns closely with Bock et al.’s (2004) observation that habitual use of preferred expression types shapes attentional priorities during time-telling tasks. For example, U.S.-American English speakers, who predominantly rely on absolute expressions, tend to fixate on the hour region first, even when producing relative expressions with divergent structures. Similarly, German, and possibly Czech speakers following previously reported patterns (Panfilova & Mertins, 2026), may exhibit distributed fixation patterns across both AoIs when engaging with less dominant absolute forms. These findings indicate that deviations from habitual linguistic preferences not only affect expression-specific gaze behavior but also modulate visual attention strategies when interacting with less familiar linguistic structures.

A noteworthy deviation from expected patterns emerged with Czech speakers regarding their nearly equal use of absolute and relative expressions in the present study, contrasting sharply with Panfilova and Mertins (2026) showing a stronger preference for relative expressions. One possible explanation involves participant demographics: Czech participants in previous study were notably older (mean age = 31) than those tested in the present experiment (mean age = 22.7), whereas German groups in both studies are well comparable: 25 in Panfilova and Mertins (2026) vs. 23 in the present study. This generational shift may reflect differences in exposure to analog clocks, as younger adults tend to rely more on digital devices for time reading, potentially reducing reliance on spatially based relative formulations. Another contributing factor may involve stimulus characteristics rather than participant intent: the inclusion of irregular times such as 12:13 may have encouraged Czech participants to produce more absolute forms (*dvanáct třináct*, ‘twelve thirteen’), whereas German speakers tended to round the irregular times, producing expressions such as *fast Viertel nach Zwölf* (‘almost quarter past twelve’). Despite producing more absolute expressions than expected, Czech speakers exhibited fixation behaviors similar to German participants, showing no clear preference for either AoI when producing absolute expressions. This suggests that habitual linguistic preferences for relative expressions persist at an attentional level even when expression choice changes due to stimulus properties. Future research should further examine how experimental context interacts with linguistic preferences to shape both linguistic behavior and visual attention strategies across different speaker groups.

The questionnaire results on clock-usage habits showed that, across all three language groups, the vast majority of participants primarily relied on digital devices, such as smartphones or smartwatches, to read time (German = 88%, Czech = 83%, Russian = 92%), whereas only a small minority reported analog or mixed usage. Because this pattern revealed minimal variance across groups, no inferential statistical analyses could be conducted. This outcome supports previous reports of declining interaction with analog displays among younger generations (McDaniel et al., 2024) but also suggests that age is not the only relevant factor. The older mean age of Russian-speaking group indicates that additional influences, such as technological access or daily time‑reading practices, may contribute to reduced use of analog clocks across all language groups. These findings underscore the need for a more detailed survey design capturing such variables in future research. Future studies should include larger and more demographically diverse samples to examine whether differences in exposure to analog clocks may influence linguistic preferences in time‑telling.

While our study provides valuable insights into cross-linguistic differences in gaze behavior during time-telling tasks, several limitations should be acknowledged. First, demographic characteristics differed across groups. The Russian‑speaking sample showed a broader age range and more heterogeneous educational backgrounds than the German and Czech student samples. Furthermore, most participants had recently moved to a German‑speaking environment as refugees and were predominantly Russian-Ukrainian bilinguals. To examine possible differential age effects across language groups, we extended the model including an Age * Language interaction. This interaction did not improve model fit and was non‑significant, suggesting that age did not differentially modulate fixation patterns across groups. Regarding educational background, the majority of Russian-speaking participants (30 of 37) either held or were enrolled in higher education programs. Finally, with respect to bilingualism, all Russian‑speaking participants reported Russian as their native language used at home; moreover, Russian and Ukrainian share identical structural means for expressing time relations. Nevertheless, future research should recruit more strictly age‑matched and socio‑demographically comparable samples across languages to further strengthen cross‑linguistic generalizability. Second, the experiment was conducted in a laboratory setting in a single session of about thirty minutes, which may have affected participants’ engagement and sustained attention, particularly toward the end of the task. Third, potential frequency‑based preferences for certain time expressions at specific clock positions were not explicitly controlled. In everyday language use, times near full hours and conventional reference points (quarter, half, three‑quarters) are typically expressed relatively rather than absolutely, and the structure of such reference points differs across the languages investigated (see Panfilova & Mertins, 2026). Some stimuli may therefore have elicited stronger biases toward relative expressions due to their position on the clock face. Future research should consider balancing stimuli across these high‑frequency regions to disentangle structural and frequency‑driven effects on time‑expression production and associated gaze patterns.

Our findings have a number of implications. They underscore how tightly integrated linguistic preferences are with cognitive processes like visual attention, even within relatively simple communicative contexts like reading clocks. Cross-linguistic comparisons reveal important variability in how different speaker groups process identical stimuli based not only on syntax but also on broader linguistic preferences. The observed flexibility among Russian participants highlights potential avenues for exploring individual variation within multilingual populations where competing structural norms coexist. From an applied perspective, these insights could inform language teaching practices by emphasizing connections between grammatical structures and cognitive strategies.

Further research incorporating additional languages or bilingual populations would help clarify whether observed trends generalize beyond Germanic-Slavic contexts. Future work might explore whether similar effects occur under different task conditions, for example, using digital clocks exclusively instead of analog displays, or examining real-world scenarios where contextual ambiguity requires spontaneous selection between expression types without explicit instructions.

By examining how initial fixations align with linguistic output across Germanic-Slavic contexts, this study provides new evidence for the dynamic interplay between language-specific preferences and cognitive processes such as visual attention during speech production involving temporal information.

## Data Availability

The data and analysis for this study have been registered on the Open Science Framework (OSF) at https://osf.io/ury9d/overview?view_only=307304540d23487bac594cf3263f35fa and will be made publicly available following publication.
